# Cost-effectiveness of adherence therapy versus health education for people with schizophrenia: randomised controlled trial in four European countries

**DOI:** 10.1186/1478-7547-11-12

**Published:** 2013-05-25

**Authors:** Anita Patel, Paul McCrone, Morven Leese, Francesco Amaddeo, Michele Tansella, Reinhold Kilian, Matthias Angermeyer, Martijn Kikkert, Aart Schene, Martin Knapp

**Affiliations:** 1 Centre for the Economics of Mental & Physical Health, PO 24, Institute of Psychiatry, King’s College London, De Crespigny Park, London, SE5 8AF, United Kingdom; 2 Health Service & Population Research Department, PO 29, Institute of Psychiatry, King’s College London, De Crespigny Park, London, SE5 8AF, United Kingdom; 3 Department of Public Health and Community Medicine, Section of Psychiatry and Clinical Psychology, University of Verona, Policlinico G.B. Rossi, Piazzale L.A. Scuro 10, Verona, 37134, Italy; 4 WHO Collaborating Centre for Research and Training in Mental Health and Service Evaluation, University of Verona, Policlinico G.B. Rossi, Piazzale L.A. Scuro 10, Verona, 37134, Italy; 5 Department of Psychiatry and Psychotherapy, Ulm University, am Bezirkskrankenhaus Günzburg, Ludwig-Heilmeyer-Str. 2, Günzburg, D-89312, Germany; 6 Center for Public Mental Health, Untere Zeile 13, Gösing am Wagram, A-3482, Austria; 7 Arkin, Overschiestraat 65, Amsterdam, 1062 XD, The Netherlands; 8 Department of Psychiatry, Academic Medical Center, University of Amsterdam, Meibergdreef 5, Room PA1-156 1105 AZ, Amsterdam, The Netherlands; 9 Personal Social Services Research Unit, London School of Economics & Political Science, Houghton Street, London, WC2A 2AE, United Kingdom

**Keywords:** Cost effectiveness, Quality-adjusted life year, Antipsychotic, Adherence, Schizophrenia, Psychological therapy

## Abstract

**Background:**

Non-adherence to anti-psychotics is common, expensive and affects recovery. We therefore examine the cost-effectiveness of adherence therapy for people with schizophrenia by multi-centre randomised trial in Amsterdam, London, Leipzig and Verona.

**Methods:**

Participants received 8 sessions of adherence therapy or health education. We measured lost productivity and use of health/social care, criminal justice system and informal care at baseline and one year to estimate and compare mean total costs from health/social care and societal perspectives. Outcomes were the Short Form 36 (SF-36) mental component score (MCS) and quality-adjusted life years (QALYs) gained (SF-36 and EuroQoL 5 dimension (EQ5D)). Cost-effectiveness was examined for all cost and outcome combinations using cost-effectiveness acceptability curves (CEACs).

**Results:**

409 participants were recruited. There were no cost or outcome differences between adherence therapy and health education. The probability of adherence therapy being cost-effective compared to health education was between 0.3 and 0.6 for the six cost-outcome combinations at the willingness to pay thresholds we examined.

**Conclusions:**

Adherence therapy appears equivalent to health education. It is unclear whether it would have performed differently against a treatment as usual control, whether such an intervention can impact on quality of life in the short-term, or whether it is likely to be cost-effective in some sites but not others.

**Trial registration:**

Trial registration: Current Controlled Trials ISRCTN01816159

## Background

Schizophrenia has notable impacts upon patients, their families, services and the wider economy [1]. Due to its chronic nature, the main aim of health and social care interventions is to improve symptoms, long-term health and quality of life. Treatments and services come at a considerable cost and although it is entirely appropriate to invest resources in helping those affected to manage their illness, there are inevitable pressures to contain costs and use budgets as effectively as possible. Non-adherence (or non-compliance) with anti-psychotic medication is common, due to the severe side-effects that are associated with many of them, and is associated with higher inpatient and total treatment costs [2]. Improving medication adherence is therefore a potential avenue for achieving savings in health care expenditure.

One potential approach is adherence therapy. It mainly uses educational, cognitive-behavioural or motivational techniques to encourage people with schizophrenia to adhere to their prescribed medication regime. There is clearly a need for further discussion and research about the effectiveness, let alone cost-effectiveness, of such treatments as indicated by a recent questioning of the issuing and interpretation of National Institute for Health and Care Excellence (NICE) guidance in England on this matter [3]. In addition to general budget constraints, there can also be resource constraints such as the relative lack of professionals trained in delivering such therapies. Therefore, as well as exploring whether such therapies can avoid unnecessary health care and other costs, there is the additional economic dimension of exploring how these scarce psychological treatments should be allocated.

We examined an adherence therapy as part of a large multi-centre randomised controlled trial (Quality of Life following Adherence Therapy for People Disabled by Schizophrenia and their Carers; QUATRO). Effectiveness evidence from that trial suggested that adherence therapy was equivalent to health education in improving quality of life [4]. It is now widely recognised that health care decision-making should move away from inference based on statistical significance [5] to avoid what Claxton et al. [6] describe as the perverse (and costly) situation of selecting a technology with the lowest chance of being cost-effective. We therefore examined the cost-effectiveness of adherence therapy using a decision-making framework which incorporates any uncertainty surrounding cost and outcomes data.

## Methods

Full details of the trial have been described by Gray et al. [4] To summarise, 409 participants with (a) a clinical and research diagnosis of schizophrenia, (b) a need for continuing anti-psychotic medication for at least a year following baseline and (c) evidence of clinical instability in the year before baseline were recruited between June 2002 and October 2003 from a range of general adult psychiatric inpatient and community services at 4 centres: Amsterdam in the Netherlands; Croydon (hereafter referred to as London) in England; Leipzig in Germany; and Verona in Italy. Ethical approval was obtained by all relevant local research ethics committees and participants gave written informed consent: Institute of Psychiatry Research Ethics Committee.

### Interventions

Participants were randomised to receive either adherence therapy or a health education package. Adherence therapy was a pragmatic intervention based on motivational interviewing and cognitive behavioural techniques, and aimed to achieve concordance about medication between the participant and therapist. It consisted of five key interventions: medication problem solving; a medication timeline; exploring ambivalence about medication; discussing beliefs and concerns about medication; and using medication in the future. The control intervention, a health education package, aimed to control for the time and non-specific effects of a therapeutic relationship. It provided information on a range of health education topics (e.g. physical health, diet and health and safety in the home), presented in a didactic way without any adherence therapy techniques to clearly differentiate it from the adherence therapy. Both interventions were delivered in routine clinical settings to maximise generalisability. Treatment completion was defined as attendance of at least 5 out of 8 sessions (each lasting 30–50 minutes) over a maximum five-month period.

### Data collection

Comprehensive data were collected on all health, social care and other relevant services used by individual study members using a tailored version of the Client Socio-demographic & Service Receipt Inventory (CSSRI-EU). This was adapted from a version specifically developed (with local language equivalents) for another European study [7] and covered: socio-demographics; living situation/accommodation; education, employment and income; time off work; use of health, social care and criminal justice system resources; and informal care. It was administered by face-to-face interview with participants (supplemented with information from key workers and service providers where necessary) at baseline and at one year follow-up, each time covering resource use for the previous 3 months, except in the case of inpatient stays, which were measured for the previous 12 months.

Resources related to the interventions were measured in terms of the number of sessions attended by each participant, the duration of each session and the non-contact time spent by the therapist to prepare or follow-up a session. Therapists extracted these data from their patient case notes onto a study proforma.

### Costs

Individual-level costs were calculated by multiplying resource use quantities with country-specific unit costs (Table 1). These were best available estimates from local or national data for each country, based on guidelines tested previously [8]. Alternative approaches were taken for some resource costs. Firstly, some specialised accommodation unit costs were obtained directly from accommodation providers. Secondly, as it was infeasible to collate country-specific unit costs for each of the huge range of medications that patients were likely to report, British medication unit costs were applied to all data using gross domestic product purchasing power parities (GDP PPPs) [9] to adjust for price levels in each country. Finally, the same approach was taken for criminal justice system services given that relatively few people were expected to use these services, such the costs were expected to contribute little to total costs and relevant cost data were not readily available. Inevitably we could not locate/calculate some unit costs within the available time and resources and we imputed these by calculating ratios of one unit cost against another within each site to account for differences in relative prices and then applying the average ratio across sites for the relevant service. Reference services against which other services were compared was selected on the basis of being in the same service sector and all sites having a unit cost estimate for it. Thus, all accommodation and hospital unit costs were compared against the unit cost for an acute psychiatric ward inpatient day, unit costs for community-based services were compared against the unit cost for a community mental health centre and unit costs for all community-based professionals were compared against the unit cost of a psychiatrist. Further details of unit cost sources and assumptions are available in Patel [10] or from the corresponding author.

**Table 1:** Unit costs in PPP-adjusted Euros (full details of sources and assumptions are available upon request from the corresponding author)

**Item**	**Unit**	**Amsterdam**	**Leipzig**	**London**	**Verona**
**Interventions**					
Adherence therapy & health education	Therapist hour	43.16	22.36	31.62	20.82
**Earnings**					
National average wage	Day	115.85	136.23	114.71	129.46
**Accommodation**					
Overnight facility, 24 hours staffed	Day	**156.07**	58.21	80.07	109.77
Overnight facility, staffed (not 24 hours)	Day	**70.94**	58.21	15.20	17.66
Overnight facility, unstaffed	Day	**28.38**	**10.68**	15.20	NA
**Hospital inpatient services**					
Acute psychiatric ward	Inpatient day	472.95	178.51	262.52	312.59
Psychiatric rehabilitation ward	Inpatient day	197.06	178.51	262.52	**253.20**
Long-stay ward	Inpatient day	75.22	178.51	200.08	**168.80**
Emergency/crisis centre	Inpatient day	**718.88**	178.51	536.38	**475.14**
General medical ward	Inpatient day	273.75	253.64	397.79	288.91
**Hospital outpatient services**					
Psychiatric outpatients	Attendance	49.14	**46.41**	137.64	56.28
Non-psychiatric outpatients	Attendance	49.68	**39.27**	133.39	13.71
Day hospital	Attendance	137.90	42.46	106.37	**96.90**
**Community-based services**					
Community mental health centre	Minute	0.29	0.43	1.39	0.10
Day care centre	Minute	0.29	**0.19**	0.20	0.27
Group therapy	Minute	**0.33**	**0.50**	0.23	0.23
Sheltered workshop	Minute	**2.31**	**3.45**	0.17	1.68
Specialist education	Minute	**0.05**	**0.07**	0.24	**0.02**
**Community-based professionals**					
Psychiatrist	Minute	1.10	1.22	4.97	0.98
Psychologist	Minute	0.71	0.53	1.56	0.66
Primary care physician	Minute	0.90	2.55	2.55	2.22
District nurse	Minute	0.43	0.49	1.09	0.50
Community psychiatric nurse/case manager	Minute	0.53	0.48	1.46	0.50
Social worker	Minute	0.53	0.47	2.20	0.37
Occupational therapist	Minute	0.53	0.47	0.88	**0.34**
Home help/care worker	Minute	0.32	0.54	0.24	0.30
Criminal justice services*					
Police	Contact	119.08	119.04	83.93	119.17
Police cell or prison	Night	28.97	28.96	20.42	28.99
Psychiatric assessment in custody	Assessment	422.78	422.65	297.99	423.10
Criminal court	Proceeding	1999.05	1998.46	1409.01	2000.57
Civil court	Proceeding	1286.61	1286.22	906.85	1287.58
Medications*					
Range for all used	100 milligrams	0.01 to 726.73	0.01 to 726.52	0.01 to 512.23	0.01 to 727.28

Shaded areas represent imputed unit costs.

*UK unit costs converted into PPP-adjusted Euros.

Costs of adherence therapy and health education were estimated by first calculating a cost per therapist hour for each site; this was identical for both interventions because they were delivered by the same staff, but variable between sites due to differing staff mixes (nurses versus psychologists) and grades to deliver the interventions. Individual-level intervention costs were then computed by multiplying this with contact and non-contact time.

All costs were originally estimated at 2003 price levels (the most recent study year for which financial information was expected to be available at the time of unit cost data collection). Where necessary, information from the next most recent financial year was adjusted using country-specific GDP inflation rates [11]. Discounting was unnecessary as costs were only assessed for one year.

Once costs for all resource items were estimated for each participant, local cost values were converted into a common currency, Euros, for the purpose of pooled analyses, using the following GDP PPP [9] conversion rates:

▪ 1 Dutch Euro = 0.952 PPP-adjusted Euros

▪ 1 German Euro = 0.924 PPP-adjusted Euros

▪ 1 UK pound sterling = 1.419 PPP-adjusted Euros

▪ 1 Italian Euro = 1.044 PPP-adjusted Euros

All costs reported here were subsequently inflated to 2011 prices using country-specific GDP inflation rates [12].

As inpatient data were reported for a one-year period while other resource use data were reported for a 3-month period (to improve accuracy), costs for the latter were extrapolated (multiplied by 4) to also represent a one-year period.

### Outcomes

We focused on two outcomes for the economic evaluation. Firstly, the trial’s primary outcome measure, the mental component summary score (MCS) of the Medical Outcome Study 36 Item Short Form Health Survey (SF-36) [13] at one year. Secondly, quality-adjusted life year (QALY) gains over one year. We have previously reported that for this patient group, QALYs generated from the SF-36 have better distributional properties than those generated from the EuroQol 5-dimensional health state measure (EQ-5D) [14,15] so we focused on SF-36 derived QALYs and used the EQ-5D in sensitive analyses. Utility weights for each measure [16,17] were attached to health states at baseline and one year to calculate QALYs using the total area under the curve approach with linear interpolation between assessment points (and baseline adjustment for comparisons) [18]. All participant-reported outcome assessments were undertaken face-to-face at baseline (prior to randomisation) and at one year follow-up by assessors blinded to participants’ group allocation.

### Cost-effectiveness and cost-utility analyses

We took two cost perspectives: (1) health and social care and (2) societal. With two outcomes, plus an additional sensitivity analysis using the EQ5D, there were six cost-outcome combinations to link and examine.

First, we planned to calculate incremental cost-effectiveness ratios (ICERs; mean cost difference divided by mean outcome difference) for any combination showing adherence therapy group to have both higher costs and better outcomes.

Second, given difficulties around estimating confidence intervals for ICERs and the potential for error in decision-making based on statistical significance, we explored uncertainty using a cost-effectiveness plane and cost-effectiveness acceptability curves (CEACs) based on the net-benefit approach [19].

A cost-effectiveness plane represents the additional costs and additional outcomes of one intervention against another. The location of a coordinate represents which of four possible cost-effectiveness scenarios the results fall into. We constructed a plane using bootstrapped regressions (1000 replications) of study group upon total health and social care costs and SF-36-based QALYs, with covariates for baseline costs and utility respectively. The resulting coefficients of group differences were saved and plotted using a scatter graph.

CEACs represent the probability that one intervention is cost-effective compared to another, accounting for hypothetical monetary values that decision-makers may place on point improvements in each outcome. CEACs were constructed by first calculating a series of net benefits for each individual, using the following formula, where λ represents how much value a decision-maker may place on one additional unit of outcome:

$$\mathrm{Net}\;\mathrm{benefit}=\left(\lambda \;\times \;\mathrm{outcome}\right)-\;\mathrm{cost}$$

We did this for λ values ranging between 0 to 50,000 Euros (in 10,000 Euro increments). Then, for each λ value, we calculated differences in mean net benefits between the two groups using non-parametric bootstrap linear regressions (1000 repetitions) which included covariates for the baseline values of the same cost category and outcome. Finally (again for each λ value), we counted the proportion of times the adherence therapy group had a greater net benefit than the health education group and plotted these proportions as a CEAC for each cost-outcome combination.

### Analyses

Analyses were done in SPSS Version 12.0.1 [20] or Stata Version 8.2 [21]. Costs and outcomes are presented as mean values with standard deviations. Mean differences and 95% confidence intervals (CIs) were obtained by non-parametric bootstrap regressions (1000 repetitions) to account for the non-normal distribution commonly found in economic data. Although this was a randomised controlled trial and participants in both groups were expected to be balanced at baseline, baseline costs and outcomes could be predictors of follow-up values. To provide more relevant treatment-effect estimates [22], baseline costs and outcomes were added as covariates for the calculation of mean differences in costs and outcomes respectively (for the ICERs) and mean differences in net benefits (for the CEACs).

All participants were analysed according to the group to which they were randomised. Those lost to follow-up at 12 months were excluded from all analyses; at baseline, these did not differ on the three outcome measures but they had higher health and social care costs (mean difference 18,152 Euros; 95% confidence interval: 1,669 to 39,646 Euros).

Missing items for the SF-36 MCS were dealt with as per the instrument’s instructions. We excluded cases with missing items on the SF-36 and EQ5D for the purpose of utility calculations. There was relatively little item non-response on the CSSRI-EU. Where this did occur, missing values were imputed to enable calculation of total costs for as many participants as possible. A value of zero was assumed where there was no indication of whether or not the resource was used. Where there was incomplete indication of use of a resource (e.g. either number or duration of contacts was given, but not both), missing details were imputed using within-country, within-group median values for resource users with relevant data. In a few situations where there were no such valid cases from which to impute we imputed using cross-country within-group median values or, failing that, cross-country cross-group medians.

Data on health and social care service use were relatively complete (less than 2% missing for any particular service at baseline and less than 3% at follow-up). The majority of missing data occurred for two items – informal care and medications. Up to 43 (11%) participants at baseline and 42 (11%) at follow-up reported receiving help with at least one of the five categories of informal care but did not provide number of hours. For medications, between 9 and 43 (2% to 11%) participants at baseline and between 2 and 27 (1% to 7%) participants at follow-up had some data to indicate medication use, but not enough to allow precise cost estimation. Where medication name was available, costs were imputed from available estimates for those study participants taking the same medication. Where medication name was unavailable, imputations were based on overall medication cost data.

When calculating the costs of the adherence therapy and health education interventions, we summed two separate components, contact time and non-contact time. Where there was an indication of the participant attending at least one intervention session missing components were imputed using within-country, within-group median costs for those who with data.

## Results

### Participant characteristics

The sample were fairly typical of those seen in prevalence studies of schizophrenia - a mean age of 42 years, 60% male, 15% living with a partner, 40% living alone and 15% employed - and were balanced between randomisation groups (see Gray et al. [4] for further details).

### Resource use

The data demonstrate the wide-ranging resource impacts typically associated with schizophrenia. The most heavily used services at both baseline and follow-up were: psychiatric inpatient stays; psychiatric outpatient visits; community mental health centre attendances; psychiatrist contacts; primary care physician contacts; and community psychiatric nurse/case manager contacts (Table 2). At both assessments, virtually all participants reported using one of the five classes of mental health medications assessed for the study (anti-psychotics, anti-depressants, benzodiazepines, mood stabilisers and anti-cholinergics) and most had received anti-psychotics, the mainstay of schizophrenia treatment. Use of benzodiazepines appeared to be reduced in both groups at follow-up, compared with baseline (41% and 35% in each group respectively at baseline, and down to 28% in both groups at follow-up).

**Table 2:** Inputs related to adherence therapy and health education

	**Adherence therapy (n = 204)**		**Health education (n = 205)**	
	**Valid n**	**Mean**	**Valid n**	**Mean**
Number of sessions	186	7	183	6
Session duration (minutes)	173	37	173	31
Total non-contact time across all sessions for attenders (minutes)	143	91	154	81
Total non-contact time across all sessions for non-attenders (minutes)	2	10	5	5

Approximately half of each group received informal care from family and friends at baseline, mainly in the form of help in and around the house and help with activities outside of the home. Informal care receipt fell to 36% at follow-up in both groups, although weekly average hours of care received (among users) differed between the groups (26 in the adherence therapy group and 8 in the health education group). Rates of contact with criminal justice system services were low.

### Employment and time off work

Few people were employed. At baseline, four people in the adherence therapy group and three in the health education group were in voluntary employment at the time of assessment. Twenty-one per cent (n = 42 and n = 43 in the adherence therapy and health education groups respectively) were in paid, self-, sheltered or other employment. Nine per cent of participants in each group had taken time off work due to illness in the past 3 months, totalling a mean (among those who took time off work) of 34 days (SD = 36) in the adherence therapy group and 27 days (SD = 32) in the health education group.

At follow-up, three people in the adherence therapy group and six in the health education group were in voluntary work. Employment rates (for paid, self, sheltered or other work) were 16% (n = 28) in the adherence therapy group and 20% (n = 40) in the health education group. Four per cent (n = 7) of the adherence therapy group took an average of 21 days (SD = 31) due to illness in the past three months, while 7% (n = 14) in the health education group took an average of 18 days (SD = 24) days off.

### Interventions

Participants in each group attended an average of 7 adherence therapy sessions and 6 health education sessions respectively (Table 3). There were more treatment completers in the adherence therapy group; 17 (9%) attended four or less sessions (in fact, 5 attended none) and thus did not meet treatment completion criteria. The health education group had 37 (20%) non-completers, of whom 9 (4.9%) attended none. Adherence therapy sessions were on average 6 minutes longer (95% confidence interval: 4 to 8); there were differences in non-contact time.

### Costs

Average costs of adherence therapy and health education interventions were 192 PPP-adjusted Euros and 138 PPP-adjusted Euros respectively (mean difference 54; 95% confidence interval 37, 70). While adherence therapy cost more than health education, both appear relatively inexpensive (although travel time by therapists and patients are not included).

The majority of total societal costs were formed of health and social care costs, with hospital inpatient costs being the largest contributor. While the adherence therapy group generally had lower costs than the health education group at baseline, confidence intervals did not suggest true differences (Table 4). There were no between-group differences in either total health and social care costs or societal costs at follow-up. Total costs fell from baseline in both groups; inpatient costs at follow-up were only 54–55% of those estimated at baseline.

### Outcomes

The groups were balanced on all outcome measures at baseline (Table 5). Both groups showed improvements in all outcome measures over time (untested) but there were no differences between the groups at follow-up.

### Cost-effectiveness and cost-utility

**Figure 1:**
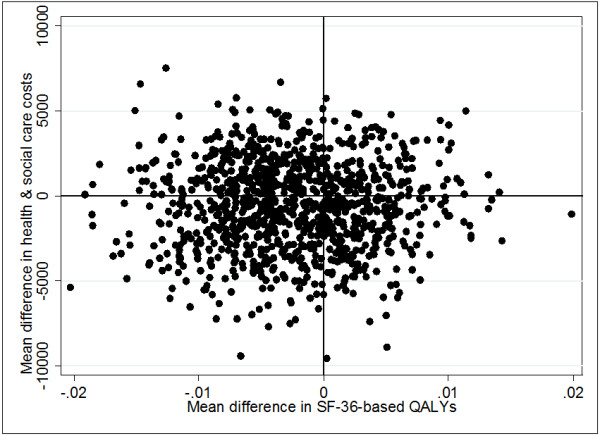
Cost-effectiveness plane (overall) of mean differences in SF36-based QALYs and mean differences in health and social care costs (PPP-adjusted Euros).

It was not necessary to calculate ICERs because none of the six cost-outcome combinations examined involved both greater costs and better outcomes for the adherence therapy group. In fact, adherence therapy may be ‘dominated’ by health education or involve lower costs alongside worse outcomes – an unlikely basis for choosing a treatment. This conclusion is supported by the spread of cost-outcome differences across all four quadrants of the cost-effectiveness plane (Figure 1) and the slight tendency for estimates to extend further across the south-west quadrant (which represents lower costs and worse outcomes).

**Figure 2:**
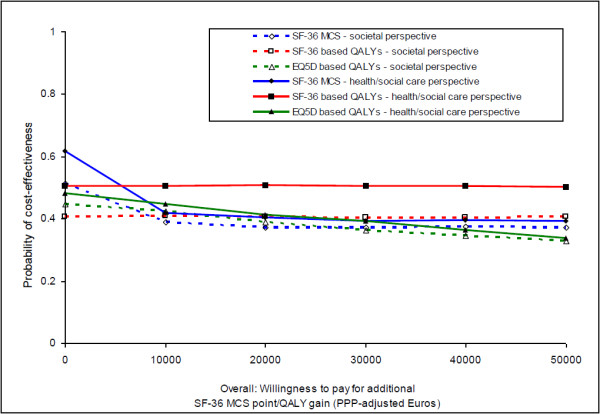
Cost-effectiveness acceptability curves (overall).

CEACs broadly confirmed the neutrality of the cost and outcome findings, with probabilities of adherence therapy being the most cost-effective option ranging between 0.3 and 0.6 from both cost perspectives and for all outcomes for the willingness to pay thresholds we examined (Figure 2). Adherence therapy had greater chances of being cost-effective from the health and social care perspective (solid lines in Figure 2) than the societal perspective (dotted lines).

**Table 3:** Resource use at baseline and 1 year follow-up (past 1 year for accommodation & inpatient services, past 3 months for all other services)

	**Adherence therapy (n = 204)**								**Health education (n = 205)**							
	**Baseline**				**1 year follow-up**				**Baseline**				**1 year follow-up**			
	**Valid n**	**Users (n, %)**		Mean*	**Valid n**	**Users (n, %)**		**Mean**^**1**^	**Valid n**	**Users (n, %)**		**Mean**^**1**^	**Valid n**	**Users (n, %)**		**Mean**^**1**^
**Specialised accommodation**	204	35	(17)	318	177	33	(19)	320	205	35	(17)	328	196	39	(20)	292
**Secondary care**																
Psychiatric inpatient days	204	86	(42)	102	176	47	(27)	113	205	83	(41)	100	196	50	(26)	100
Non-psychiatric inpatient days	204	14	(7)	16	176	6	(3)	14	205	20	(10)	40	196	12	(6)	17
Psychiatric outpatient visits	204	47	(23)	2	177	26	(15)	2	205	53	(26)	3	196	39	(20)	1
Emergency department & other outpatient visits	204	20	(10)	2	177	9	(5)	2	205	14	(7)	1	196	7	(4)	1
Day hospital visits	204	15	(7)	20	177	2	(1)	19	205	9	(4)	4	196	6	(3)	11
**Community-based services**																
Community mental health centre visits	204	54	(27)	18	177	60	(34)	19	205	49	(24)	18	196	57	(30)	17
Day care centre visits	204	23	(11)	20	177	14	(8)	16	205	18	(9)	32	196	19	(10)	21
Group therapy visits	204	3	(2)	21	177	3	(2)	21	205	4	(2)	7	196	1	(1)	12
Sheltered workshop visits	204	9	(4)	24	177	2	(1)	22	205	6	(3)	38	196	3	(2)	36
Specialist education visits	204	1	(< 1)	24	177	1	(1)	36	205	1	(< 1)	4	196	1	(1)	6
**Primary and community care professionals**																
Psychiatrist contacts	204	140	(69)	4	177	118	(37)	4	205	145	(71)	4	195	143	(73)	3
Psychologist contacts	204	3	(2)	7	177	6	(3)	15	205	8	(4)	6	196	6	(3)	14
Primary care physician contacts	204	86	(42)	2	177	80	(45)	2	205	90	(44)	3	195	84	(40)	2
District nurse contacts	204	3	(2)	27	177	3	(2)	32	205	2	(1)	7	196	4	(2)	26
Community psychiatric nurse/case manager contacts	204	71	(35)	5	177	67	(38)	8	205	77	(38)	6	195	71	(36)	11
Social worker contacts	204	30	(15)	6	177	19	(11)	4	205	20	(10)	5	195	20	(10)	4
Occupational therapist contacts	204	3	(2)	36	177	5	(3)	16	205	5	(2)	43	195	1	(1)	1
Home help/care worker contacts	204	9	(4)	24	177	5	(3)	26	205	8	(4)	19	195	10	(5)	18
**Medications**																
Antipsychotics	197	177	(90)		175	162	(93)		204	185	(91)		196	184	(94)	
Antidepressants	195	60	(31)		173	52	(30)		204	45	(22)		196	47	(24)	
Benzodiazepines	194	79	(41)		174	49	(28)		204	71	(35)		196	55	(28)	
Mood stabilisers	194	20	(10)		173	17	(10)		204	23	(11)		196	22	(11)	
Anticholinergics	195	26	(13)		173	29	(17)		204	33	(16)		196	32	(16)	
**Informal care hours per week**	204	103	(50)	11	177	63	(36)	14	205	98	(48)	12	195	69	(35)	8
**Criminal justice system**																
Police contacts	204	13	(6)	2	177	6	(3)	1	205	13	(6)	1	196	9	(5)	1
Nights spent in police cell or prison	204	3	(1)	22	177	1	(1)	1	205	3	(1)	2	196	2	(1)	6
Psychiatric assessment whilst in custody	204	2	(1)	1	177	0	-	-	205	1	(< 1)	1	196	1	(1)	1
Criminal court appearances	204	1	(< 1)	1	177	0	-	-	205	1	(< 1)	1	196	2	(1)	2
Civil court appearances	204	3	(1)	1	177	1	(1)	1	205	1	(< 1)	1	196	0	-	-

^1^. Mean for those who used services.

**Table 4:** Mean one-year costs at baseline and 1 year follow-up (PPP-adjusted Euros, 2011 prices)

	**Adherence therapy (n = 204)**			**Health education (n = 205)**			**Adherence therapy – Health education**^**1**^			
	**Valid n**	**Mean**	**(SD)**	**Valid n**	**Mean**	**(SD)**	**Unadjusted comparisons**		**Baseline-adjusted comparisons**	
							**Mean difference**	**95% confidence interval**	**Mean difference**	**95% confidence interval**
**Baseline**										
Accommodation	204	5637	(13837)	205	6676	(16508)	-1039	-4323, 1640	na	na
Inpatient services	204	13649	(30841)	205	12659	(27797)	990	-4440, 6253	na	na
Outpatient services	204	686	(2938)	205	314	(794)	**372**	**30, 884**	na	na
Community-based services	204	2973	(18688)	205	4532	(25686)	-1559	-6200, 2912	na	na
Community-based professionals	204	998	(1370)	205	1088	(2797)	-90	-555, 289	na	na
Medication	197	4400	(6387)	204	4103	(6405)	297	-1031, 1527	na	na
**Subtotal from health/social care perspective**	197	27427	(36015)	204	29484	(41535)	-2057	-9916, 5882	na	na
Informal care	204	5300	(12230)	205	5461	(14533)	-161	-2756, 2667	na	na
Time off work	202	1656	(7846)	204	1339	(6514)	316	-1190, 1631	na	na
Criminal justice system	204	442	(4524)	205	368	(4440)	74	-862, 1012	na	na
Sub-total for non-health/social care costs	202	7451	(14805)	204	7197	(16157)	253	-3093, 3273	na	na
**Total from societal perspective**	195	35190	(39442)	203	36828	(44573)	-1638	-9801, 6719	na	na
**1 year follow-up**										
Adherence therapy or health education intervention	204	192	(93)	205	138	(77)	**54**	**37, 70**		
Accommodation	177	6112	(14523)	196	6504	(15953)	-392	-3569, 2659	194	-1805, 2259
Inpatient services	176	7411	(20737)	196	6976	(22574)	435	-4025, 4732	418	-3719, 4318
Outpatient services	177	235	(832)	196	226	(613)	10	-137, 166	5	-144, 160
Community-based services	177	1350	(5108)	196	3865	(29011)	-2515	-7420, 589	-237	-2168, 1298
Community-based professionals	177	1545	(6969)	195	1473	(6737)	72	-1267, 1405	76	-1324, 1411
Medication	175	3202	(3942)	196	3549	(4737)	-347	-1285, 585	-483	-1268, 322
**Subtotal from health/social care perspective, including intervention cost**	174	20115	(28339)	195	22597	(40727)	-2483	-10017, 4448	-757	-5820, 4386
**Subtotal from health/social care perspective, excluding intervention cost**	174	19919	(28332)	195	22459	(40720)	-2540	-10075, 4385	-816	-5877, 4331
Informal care	177	4639	(17298)	194	2813	(6377)	1826	-634, 4602	1859	-611, 4532
Time off work	176	423	(3541)	196	699	(4146)	-277	-1043, 506	-320	-1063, 408
Criminal justice system	177	40	(372)	196	978	(9963)	-938	-2597, 46	-937	-2605, 46
Sub-total for non-health/social care costs	176	5118	(17679)	194	4508	(12220)	610	-2282, 3881	596	-2451, 4000
**Total from societal perspective, including intervention cost**	173	25346	(32406)	193	26787	(41743)	-1442	-9722, 6213	10	-6915, 6235
**Total from societal perspective, excluding intervention cost**	173	25149	(32404)	193	26648	(41737)	-1499	-9774, 6153	-49	-6979, 6171

^1^. Based on bootstrapped linear regression of group upon cost (1000 repetitions).

*na* = not applicable to baseline comparisons.

**Table 5:** Outcomes at baseline and 1 year follow-up

	**Adherence therapy (n = 204)**			**Health education (n = 205)**			**Adherence therapy – Health education**^**1**^			
	**Valid n**	**Mean**	**(SD)**	**Valid n**	**Mean**	**(SD)**	**Unadjusted comparisons**		**Baseline-adjusted comparisons**	
							**Mean difference**	**95% confidence interval**	**Mean difference**	**95% confidence interval**
**SF-36 MCS**										
Baseline	191	38.39	(11.22)	195	40.11	(12.15)	-1.72	-4.14, 0.54	na	na
1 year follow-up	175	40.24	(11.97)	192	41.32	(11.49)	-1.08	-3.43, 1.42	-0.33	-2.41, 1.79
**SF-36 utilities and QALYs**										
Baseline utility	191	0.66	(0.12)	192	0.68	(0.13)	-0.02	-0.04, 0.01	na	na
1 year follow-up utility	177	0.68	(0.14)	190	0.69	(0.13)	-0.01	-0.04, 0.02	-0.005	-0.03, 0.02
1 year QALY gain	166	0.67	(0.11)	179	0.68	(0.12)	-0.01	-0.04, 0.01	-0.002	-0.01, 0.01
EQ5D utilities and QALYs										
Baseline utility	196	0.67	(0.30)	198	0.69	(0.28)	-0.02	-0.08, 0.03	na	na
1 year follow-up utility	174	0.68	(0.31)	193	0.74	(0.26)	-0.06	-0.12, -0.003	-0.04	-0.09, 0.01
1 year QALY gain	170	0.67	(0.26)	188	0.72	(0.23)	-0.05	-0.10, 0.01	-0.02	-0.05, 0.01

^1^. Based on bootstrapped linear regression of group upon cost (1000 repetitions).

*na* = not applicable to baseline comparisons.

## Discussion

Effectiveness evidence from the QUATRO study suggested that adherence therapy was equivalent to health education in improving quality of life for people with schizophrenia [4]. This economic evaluation confirms this equivalence by finding no differences in costs (from either of two perspectives), quality-adjusted life years or cost-effectiveness.

As the study shows small outcome improvements and cost reductions over time in both groups, it is unclear whether either intervention had any impact at all and the changes simply reflect the natural course of participants’ recovery because they were recruited during a clinically unstable period. While controlling for the time and attention inputs was necessary, it causes difficulties for interpreting the findings. It is possible that results may have differed with a comparison of treatment as usual [3]. Although many evaluations of adherence interventions naturally assess impact on adherence to medication, the QUATRO study focused on the more over-arching outcome of quality of life (in the expectation that this could be improved via lower symptoms and better functioning) and found no such differences over one year. We do not know whether such effects are more likely to occur in the longer term or whether adherence may not be a mediating factor in improving quality of life, as also suggested in a more recent study of an adherence therapy intervention for people with psychotic disorders [23].

While there is a wealth of evidence suggesting that improvements in medication adherence are associated with reductions in readmissions (e.g. see Staring et al. [23] for a recent example), there is comparatively less by way of ‘formal’ economic evaluation of non-pharmacological interventions (see Andrews et al. [24] for a recent review). A randomised controlled trial of 74 people with psychosis about to move from inpatient residence found that those receiving compliance therapy were five times more likely than those receiving non-specific counselling to take their medication without prompting, and over an 18-month follow-up period had better global functioning, insight, adherence and attitudes to their medication [25]. The associated economic evaluation (which took a broad perspective incorporating health and social care services, education, social security and housing supports, and criminal justice contacts) found the two interventions had similar costs during each of the three 6-month follow-up phases and over the full 18 months [26]. Combined with improved outcomes, this suggested cost-effectiveness. Significant correlations were found between greater adherence and higher costs over the first six months. Therefore, improving adherence initially increased costs, although there was an offsetting reduction over time.

Our study has several strengths. It took a broad cost perspective, which is a necessity to encompass the many and broad-ranging impacts that schizophrenia incurs [27]. The study also had an exceptionally good follow-up rate with minimal missing data among those that were followed up, although it is unclear what the effect on findings may have been if those lost to follow-up had been included given that they had higher costs at baseline compared with those followed up. Finally, this multi-country economic evaluation was undertaken by applying mostly country-specific unit costs to country-specific resource use data, and conducting pooled analyses based on costs converted to a common currency using purchasing power parities. This combination of approaches had the advantages of preserving the within-country link between resource use, costs and outcomes and maintaining a large sample size (generally, but especially so in the context of schizophrenia studies).

**Figure 3:**
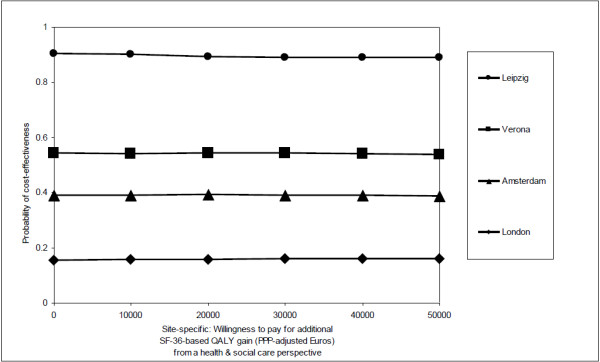
Cost-effectiveness acceptability curves (site-specific).

However, the multi-country approach also carries methodological challenges (such as wide-scale unit cost collation) and limitations for interpretation and generalisability; for example, there may be variations in adherence to anti-psychotic medication due to broad contextual factors such as culture or ethnicity [28] which in turn impact on costs [29]. We did not intend to examine costs and cost-effectiveness for each site separately because of insufficient sample size for such sub-group analyses (moving away from statistical significance towards the CEAC approach doesn’t necessarily make small sample studies acceptable [5]). Despite similarities in quality of life outcomes between sites, there were variations in resource use, unit costs and resource costs and this may affect the application of the findings to the individual study countries and for policy-making. Such observations could also arise in multi-centre studies carried out within a single country, but are more noticeable in multi-national studies perhaps simply because we are more likely to look for them in this situation. For example, in examining the relative contribution of different resources to total cost, Leipzig shows the greatest difference as compared to the other sites and the pooled results. Its specialised accommodation costs were relatively low compared to the other sites, which is probably due to few such facilities existing there. It also had higher medication costs, both in absolute terms and in terms of the proportion they contribute to total costs, which likely due to greater medication use, rather than differing unit costs, given that all medication costs were based on UK prices. Although there were no statistically significant differences in total health and social care costs between the two groups at one year follow-up at any of the individual sites, there were marked differences in the size of the observed mean (baseline-adjusted) difference: -8868 PPP-adjusted Euros in Leipzig (i.e. a cost saving in the adherence therapy group) to 5421 PPP-adjusted Euros in London (i.e. a cost saving in the health education group). The impact of such variations is apparent in site-specific CEACs (Figure 3), with probabilities of the cost-effectiveness of adherence therapy being highest in Leipzig and lowest in London, for the threshold range examined. While we are cautious about focusing on these site-specific findings due to sample size limitations, they clearly suggest that the potential value of adherence therapy varies across sites and that pooled analyses in trials may not portray such variations. This is an important finding because multi-country trials are increasingly used to increase sample size, speed up recruitment and/or increase generalisability. We further explored the impact on site-specific CEACs when costs are analysed in their local currency, rather than being standardised to a common currency, whilst being kept at their original price year of 2003, rather than being inflated to recent price levels using an inflation rate (GDP-based) that may or may not accurately reflect changes in health care costs over time. This had virtually no impact on the probabilities of cost-effectiveness for each site, with values for all threshold levels varying by no more than 0.007 points away from those obtained from inflated PPP-adjusted Euros.

## Conclusions

This study confirms the substantial costs that are required to care for people with schizophrenia and thus the need for cost-effective support for this group of people. This evaluation suggests that adherence therapy doesn’t meet that need when the focus in on quality of life. The clarity of this conclusion is affected by the unknown impact of using an active, rather than treatment as usual, control, uncertainty about the ability of such interventions to impact on quality of life in the short-term and variations in the cost-effectiveness of adherence therapy across the sites.

## Abbreviations

CEAC: Cost-effectiveness acceptability curve; CSSRI-EU: Client socio-demographic & service receipt Inventory; EQ5D: EuroQol 5-dimensional health state measure; GDP: Gross domestic product; ICER: Incremental cost-effectiveness ratio; MCS: Mental component score; NICE: National institute for health and clinical excellence; PPP: purchasing power parity; QALY: Quality-adjusted life year; QUATRO: Quality of life following adherence therapy for people disabled by Schizophrenia and their carers; SF-36: Medical outcome study 36 Item short form health survey; UK: United Kingdom

## Competing interests

The authors declare that they have no competing interests.

## Authors’ contributions

AP designed the economic evaluation, analysed the data and drafted the manuscript. PM designed the economic evaluation and commented on the manuscript. ML oversaw the statistical design of the study, advised on analysis and commented on the manuscript. FA, RK and MKi assisted with estimating unit costs for the Italian, German and Dutch components of the data respectively and commented on the manuscript. MKn, MT and AS conceived the study and participated in its overall design and coordination and commented on the manuscript. All authors read and approved the final manuscript.
